# Transcriptomic Analysis Reveals Evidence for a Cryptic Plastid in the Colpodellid *Voromonas pontica*, a Close Relative of Chromerids and Apicomplexan Parasites

**DOI:** 10.1371/journal.pone.0096258

**Published:** 2014-05-05

**Authors:** Gillian H. Gile, Claudio H. Slamovits

**Affiliations:** Department of Biochemistry and Molecular Biology, Dalhousie University, Halifax, Nova Scotia, Canada; University of Cambridge, United Kingdom

## Abstract

Colpodellids are free-living, predatory flagellates, but their close relationship to photosynthetic chromerids and plastid-bearing apicomplexan parasites suggests they were ancestrally photosynthetic. Colpodellids may therefore retain a cryptic plastid, or they may have lost their plastids entirely, like the apicomplexan *Cryptosporidium*. To find out, we generated transcriptomic data from *Voromonas pontica* ATCC 50640 and searched for homologs of genes encoding proteins known to function in the apicoplast, the non-photosynthetic plastid of apicomplexans. We found candidate genes from multiple plastid-associated pathways including iron-sulfur cluster assembly, isoprenoid biosynthesis, and tetrapyrrole biosynthesis, along with a plastid-type phosphate transporter gene. Four of these sequences include the 5′ end of the coding region and are predicted to encode a signal peptide and a transit peptide-like region. This is highly suggestive of targeting to a cryptic plastid. We also performed a taxon-rich phylogenetic analysis of small subunit ribosomal RNA sequences from colpodellids and their relatives, which suggests that photosynthesis was lost more than once in colpodellids, and independently in *V. pontica* and apicomplexans. Colpodellids therefore represent a valuable source of comparative data for understanding the process of plastid reduction in humanity's most deadly parasite.

## Introduction

Nearly all lineages of photosynthetic eukaryotes have non-photosynthetic members. The best-known examples are parasitic land plants [1], [2], but parasitic and heterotrophic green algae and parasitic red algae are also known [3]–[7]. Among secondary algal lineages, photosynthesis has been lost multiple times in cryptophytes [8] and euglenids [9], at least once in haptophytes [10], and multiple times from within the photosynthetic clade of stramenopiles [11]. Most non-photosynthetic algae are fairly young lineages, i.e. they have close photosynthetic relatives, but the apicomplexan parasites are a prominent exception. Apicomplexans have maintained a non-photosynthetic plastid (apicoplast) for 700 million years [12], [13], since their divergence from a photosynthetic ancestor they share with dinoflagellates [14].

The idea that apicomplexans and dinoflagellates share a photosynthetic ancestor was initially complicated by the fact that the deepest-branching dinoflagellates are non-photosynthetic [15] and by early analyses suggesting that apicomplexan plastids have a green, rather than a red algal origin like dinoflagellates ([16], but see [17]). The discovery of *Chromera velia* and *Vitrella brassicaformis*, close photosynthetic relatives of apicomplexans whose plastids share characteristics with both apicomplexans and dinoflagellates, has confirmed that apicomplexans and dinoflagellates share a red plastid-bearing photosynthetic ancestor [14], [18], [19]. From this perspective, the other, lesser-known descendants of this ancestor, namely colpodellids, perkinsids, and *Oxyrrhis*, might be expected to maintain a non-photosynthetic plastid. Sequence-based evidence for a cryptic plastid has been reported from *Oxyrrhis marina* [20], and a non-photosynthetic plastid has been identified by immunofluorescence in *Perkinsus marinus* [21]. On the other hand, two apicomplexan lineages (or possibly one, if gregarines and *Cryptosporidium* are sister groups [22], [23]) have lost the ancestral plastid completely [24]–[26]. To date it is unknown whether any colpodellids retain a non-photosynthetic plastid.

Colpodellids are free-living predatory flagellates that have been found inhabiting freshwater, soil, marine, and hypersaline environments [27]–[30]. They are best known as close relatives of the apicomplexan parasites and for their mode of feeding called myzocytosis, which involves puncturing the prey cell membrane and sucking out its contents. Perkinsids and certain dinoflagellates also feed by myzocytosis, hence the name Myzozoa for the phylum comprising the common ancestor of apicomplexans and dinoflagellates and all of its descendants [30]. The 11 described species of colpodellids are diverse morphologically, ranging from 5 µm to greater than 20 µm in length and having a large, hooked rostrum (*Algovora = Colpodella turpis*), a long, thin rostrum (*Chilovora = Colpodella perforans*) a small rostrum (*Colpodella angusta*) or no rostrum at all (*Colpodella gonderi*) [27]. Notable ultrastructural differences include the presence or absence of trichocysts and the organization of the subcortical alveoli as inflated and discrete, as in dinoflagellates, or flattened and fused, as in apicomplexans [27], [30]–[32]. As a result of this diversity of form, along with a paucity of readily observed morphological characters, colpodellid taxonomy has a turbulent history. Species have been described under various genera including *Colpodella*, *Spiromonas*, *Alphamonas, Dingensia*, *Nephromonas*, and *Bodo*, then united under the single genus *Colpodella* [27], only to be split again into *Algovora*, *Voromonas*, *Chilovora*, *Colpodella*, and the reinstated *Alphamonas* [30]. Similarly, molecular phylogenetic analyses of colpodellids have been conflicting or poorly supported, with colpodellids forming a sister clade to apicomplexans [33], [34] or branching separately at the base of dinoflagellates and/or apicomplexans depending on the methods used [30].

In order to determine whether colpodellids might harbor a cryptic plastid, we searched transcriptomic data from *V. pontica* for genes encoding homologs of apicoplast biosynthetic enzymes. The metabolic functions of the apicoplast are well understood thanks to efforts to develop new antimalarial drugs. Iron sulfur cluster, heme, fatty acid, and isoprenoid biosynthesis are perhaps the best-studied apicoplast metabolic pathways, and several transporters and other enzymes are known [35]–[38]. In order to provide a phylogenetic framework against which to interpret these data, we also performed a deeply sampled phylogenetic analysis including many recently available environmental sequences. Together these comparative genomic and phylogenetic analyses aim to provide an evolutionary perspective on the process of converting a photosynthetic plastid into a reduced, non-photosynthetic apicoplast.

## Results

### Voromonas pontica transcriptome

Here we report transcriptomic data from the marine, predatory protist *V. pontica*, the first transcriptomic data reported for any colpodellid. To generate the final transcriptome dataset, a filtering step was necessary to remove sequences from *Percolomonas cosmopolitus*, the eukaryotic prey of *V. pontica*. We established a single eukaryote *P. cosmopolitus* culture by serial dilution of the mixed predator/prey culture (ATCC 50640), sequenced total cDNA from *P. cosmopolitus* and from the mixed culture, trimmed and assembled reads from each, then bioinformatically subtracted *P. cosmopolitus*-like sequences (90% nucleotide identity for 90% of the contig length) from the raw mixed culture contigs (see Materials and Methods for further details). The resulting *V. pontica* transcriptome consists of 13,970 contigs including splicing variants (11,049 unique loci) predicted by Trinity [39] with median and mean contig lengths of 508 and 702 bp, respectively, suggesting incomplete read coverage for many transcripts. For an additional, independent, rough estimate of completeness, we used CEGMA [40], [41] to determine the proportion of a conserved eukaryotic core gene set that is present in our dataset. Using an expect value of e^−10^ and coverage of the HMMER profile set to 70%, CEGMA found 60% of its core eukaryotic genes in our dataset. For 50% HMMER profile coverage, the proportion increased to 70%, reinforcing the idea that many of our contigs are incomplete relative to the mRNAs from which they are derived, and suggesting that a sizeable proportion of expressed genes are not represented.

### Phylogenetic position of *V. pontica*

**Figure 1:**
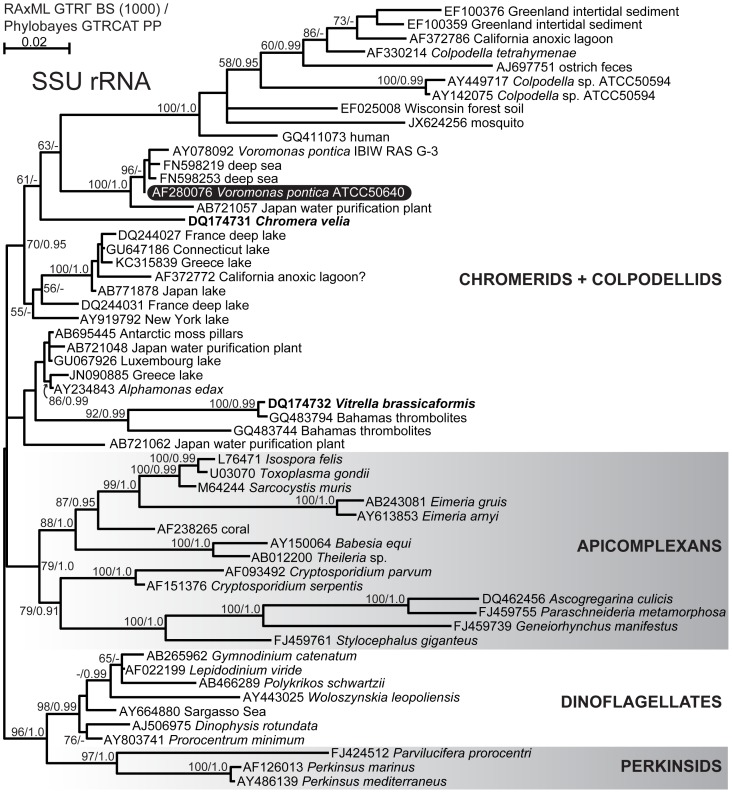
Bayesian consensus phylogeny of small subunit rRNA sequences from alveolates representing the available diversity of colpodellids, chromerids, and related environmental sequences. Support for nodes is indicated by % bootstrap support (out of 1000) in the ML analysis (RAxML GTRΓ)/Bayesian posterior probability (Phylobayes GTRCAT) where greater than 55 or 0.9. The subject of this study, *Voromonas pontica*, is indicated by white text on a black background. The photosynthetic chromerids *Chromera velia* and *Vitrella brassicaformis* are indicated by bold text. A question mark after accession AF372772 indicates a possible misidentification or chimeric sequence; this study also sampled a lake.

Only a handful of published phylogenetic analyses have included colpodellids to date, and many new related environmental sequences have become available since the most recent analysis. In order to provide a new and improved evolutionary framework against which to interpret our data, we assembled a dataset of small subunit ribosomal RNA (SSU rRNA) sequences spanning the phylogenetic diversity of apicomplexans and dinoflagellates, and comprehensively sampling all available colpodellid, chromerid, and related environmental sequences. We used Bayesian and maximum-likelihood (ML) methods to compute the phylogenies. In the better-resolved Bayesian tree (Fig. 1), apicomplexans and dinoflagellates form supported clades, but the relationships among colpodellids and chromerids are not well resolved. Instead, chromerids and colpodellids, neither of which are monophyletic, fall into two main clades that form a tritomy with the apicomplexan clade. In the ML topology (Fig. S1), apicomplexans and dinoflagellates likewise form supported clades, but *V. brassicaformis* and its related sequences branch most closely to apicomplexans, followed by the *Alphamonas edax* clade, though neither grouping is supported. In both ML and Bayesian analyses, however, two clades of colpodellids are recovered with strong support: *V. pontica* and its closely related marine environmental sequences, and *Colpodella tetrahymenae* and its more diverse family of colpodellid sequences from soils, intertidal sediments, and animals, including a human [42]. These two clades are united in turn by moderate support (i.e. 63% ML bootstrap, 0.87 Bayesian posterior probability) in both analyses.

### Search for plastid-associated genes

We assembled query files of enzymes from apicomplexan plastid-associated biosynthetic pathways and used tBLASTn to search the *V. pontica* filtered contigs. For a list of enzymes sought from each of the SUF iron-sulfur biosynthesis, MEP isoprenoid biosynthesis, FASII fatty acids biosynthesis, and heme biosynthesis pathways, see Table 1. This strategy resulted in the identification of seven genes putatively encoding proteins from three key plastid-associated pathways, namely SufB, SufS, DXS, IspE, IspG, ALAS and ALAD (Table 1), though note that ALAS is not a plastid-associated protein. We also searched for homologs of experimentally localized apicoplast proteins downloaded from ApiLoc v. 3 (http://apiloc.biochem.unimelb.edu.au/apiloc), and in addition to ALAD previously identified during our pathway search, we found a homolog of the apicoplast triosephosphate transporter (pPT). We recovered the full-length genes for SufB and IspE, about three quarters of the length of the genes for IspG and ALAS, and roughly half of the protein coding region for pPT. For the remaining three genes, only a small fraction was recovered (<25%) but they were nonetheless confidently recognized according to phylogenetic analyses (Figs. S3, S4, S8). We were able to obtain the complete 5′ end of the transcripts for all but SufS, DXS, and pPT (Table 1).

**Table 1:** Presence/absence of plastid-associated biosynthetic pathway enzymes sought in the *V. pontica* transcriptome.

Enzyme	Full name	5' end?	3' end?	% of protein represented
*Fe-S cluster biosynthesis*				
SufA	iron-sulfur assembly scaffold protein			
SufB	subunit of ATP-binding SufBCD complex	yes	yes	100
SufC	subunit of ATP-binding SufBCD complex			
SufD	subunit of ATP-binding SufBCD complex			
SufE	sulfur acceptor protein			
SufS	cysteine desulfurase	no	no	20
Nfu1	iron-sulfur cluster scaffold protein			
Fd	ferredoxin			
FNR	ferredoxin-NADP reductase			
*isoprenoid biosynthesis (MEP pathway)*				
DXS	1-deoxy-D-xylulose-5-phosphate (DXP) synthase	no	no	12
IspC (DXR)	DXP reductoisomerase			
IspD	2-*C*-methyl-D-erythritol 4-phosphate (MEP) cytidylyltransferase			
IspE	4-diphosphocytidyl methylerithrytol kinase	yes	yes	100
IspF	methylerythritol 2,4-cyclodiphosphate synthase			
IspG (GcpE)	1-hydroxy-2-methyl-2-butenyl 4-diphosphate (HMBPP) synthase	yes	no	75
IspH (LytB)	HMBPP reductase			
*heme biosynthesis (C4 pathway)*				
ALAS*	δ-aminolevulinic acid (ALA) synthase	yes	no	75
ALAD	ALA dehydrogenase	yes	no	25
PBGD	porphobilinogen deaminase			
UROS	uroporphyrinogen synthase			
UROD	uroporphyrinogen dehydratase			
CPOX*	coproporphyrinogen oxidase			
PPOX*	protoporphyrinogen oxidase			
FeCH*	ferrochelatase			
*fatty acid biosynthesis (FASII pathway)*				
ACP	acyl carrier protein			
FabD	malonyl-CoA:ACP transacylase			
FabG	3-ketoacyl-ACP reductase			
FabH	3-oxoacyl-ACP synthase			
FabI	enoyl-ACP reductase			
FabZ	beta-hydroxyacyl-ACP dehydratase			
GatP	glycerol-3-phosphate acyltransferase			
*transporter*				
pPT	plastidic phosphate transporter	no	no	45
*Not plastid targeted in apicomplexans.				

#### SUF genes

Our search for genes in the plastid-associated SUF iron-sulfur cluster biosynthesis pathway uncovered two candidates, SufB and SufS. For SufB, the 5′ end was complete and the 3′ end was completed by RACE, yielding a 2054 bp transcript that encodes a 555 amino acid protein. Unlike apicomplexans, whose SufB gene is encoded in the apicoplast genome, the SufB homolog in *V. pontica* carries a 20 amino acid predicted signal peptide and an additional 34 amino acids (aa) stretch before the beginning of the conserved SufB domain (Fig. S2). This 34 aa stretch is hydrophilic, rich in serine and threonine (11/34 residues) and with an excess of basic (HKR) over acidic (DE) residues (Table 2), consistent with known characteristics of plastid transit peptides [43]–[46]. For SufS, we found a 302 bp contig encoding 100 aa, or approximately 20% of the complete protein, though not the N-terminus. Apicomplexan and *P. marinus* SufS protein sequences each bear an N-terminal extension with predicted signal peptide (weakly predicted for *Plasmodium falciparum* and *P. marinus*), and are expected to be plastid-targeted.

**Table 2:** Predicted plastid targeting signal and transit peptide characteristics.

ALAD	Length (aa)	S%	H+K+R%	D+E%	SufB	Length (aa)	S%	H+K+R%	D+E%
signal peptide	17	11.8	5.9	0.0	signal peptide	20	15.0	10.0	0.0
transit peptide	62	9.7	17.7	12.9	transit peptide	33	24.2	15.2	12.1
TP first 20 aa	20	20.0	5.0	10.0	TP first 20 aa	20	25.0	25.0	5.0
mature protein	33^1^	9.1	12.1	15.2	mature protein	502	7.8	11.6	11.8
whole protein	112^1^	9.8	14.3	11.6	whole protein	555	9.0	11.7	11.3

ispE	Length (aa)	S%	H+K+R%	D+E%	ispG	Length (aa)	S%	H+K+R%	D+E%
signal peptide	20	15.0	5.0	0.0	signal peptide	22	0.0	22.7	4.5
transit peptide	83	10.8	14.4	10.8	transit peptide	50	6.0	10.0	10.0
TP first 20 aa	20	5.0	10.0	0.0	TP first 20 aa	20	5.0	10.0	0.0
mature protein	364	7.4	10.7	10.2	mature protein	403^1^	4.5	13.6	13.4
whole protein	467	8.4	11.1	9.8	whole protein	475^1^	4.4	13.7	12.6

1 protein lengths are derived from incomplete transcripts.

#### MEP genes

We found three genes encoding enzymes of the MEP pathway for isoprenoid biosynthesis, DXS, ispE, and ispG. The DXS contig, at only 247 bp (82 aa) covers approximately 12% of the predicted length of the mature protein, but nonetheless appears to be a genuine, eukaryotic DXS gene: the top blast hits are plastid-targeted DXS genes from stramenopiles, red algae, and the cryptophyte *G. theta*, sharing 70–74% amino acid sequence identity over the 82 aa query, followed by DXS from land plants and green algae, at 65–69% identity. For the ispE contig, the 5′ end of the transcript was present in the transcriptome library and we were able to complete the 3′ end by RACE. The complete 1541 bp contig encodes a 467 aa protein sharing 35–42% identity with top blast hits from algae, land plants, and chlamydiae. The ispG contig was nearly complete at the 5′ end, and we were able to complete it with 5′ RACE to yield a 1629 bp contig encoding a 475 aa predicted protein covering approximately 75% of its expected length. Again, the top blast hits are to algae and land plants, though the percent amino acid sequence identity range is 43–62%, higher than for ispE. Each of the ispE and ispG contigs encodes a putative bipartite targeting sequence at the 5′ end, consisting of a predicted typical eukaryotic signal peptide (20 and 22 aa long, respectively, Fig. S2) followed by a stretch of amino acids (83 and 50, respectively) before the start of the conserved domain (Table 2). In both cases the first 20 aa following the signal peptide carry an excess of basic (HKR) over acidic (DE) amino acid residues, resulting in an overall positive charge and consistent with other characterized plastid targeting peptides [43]–[46].

#### Heme biosynthesis genes

We found two genes for heme biosynthesis in the *V. pontica* transcriptomic data, ALAS (which is mitochondrially-targeted in other organisms) and ALAD, which is plastid-targeted in apicomplexans [93], [101]. The N-terminus of ALAS has a stretch of 105 aa before the start of sequence conservation with its homologs. This stretch may represent a mitochondrial transit peptide, though the localization predictions vary. Euk-mPLoc predicts a cytoplasmic and/or mitochondrial localization for the protein, TargetP predicts a mitochondrial protein with low confidence when the organism group is set to “plant”, but a cytoplasmic localization when “non-plant” is selected. WoLF PSORT predicts a mitochondrial localization but only when the organism group is set to “fungi”; with “plant” or “animal” selected, the top predicted location is the cytoplasm. For ALAD, the short, 454 bp contig is nevertheless complete at the 5′ end and encodes a clearly predicted signal peptide (Fig. S2) followed by a stretch of 62 aa before the start of the conserved domain. Although the first 20 aa of the putative transit peptide are unusually enriched in acidic relative to basic residues, the basic residues outnumber acidic residues when considered over the whole length of the putative transit peptide (Table 2). The encoded protein is only 25% of the expected length and exhibits 40–65% aa identity to plastid-targeted ALAD of stramenopiles, red and red-derived algae, apicomplexans, and land plants.

#### Plastidic phosphate transporter

**Figure 2:**
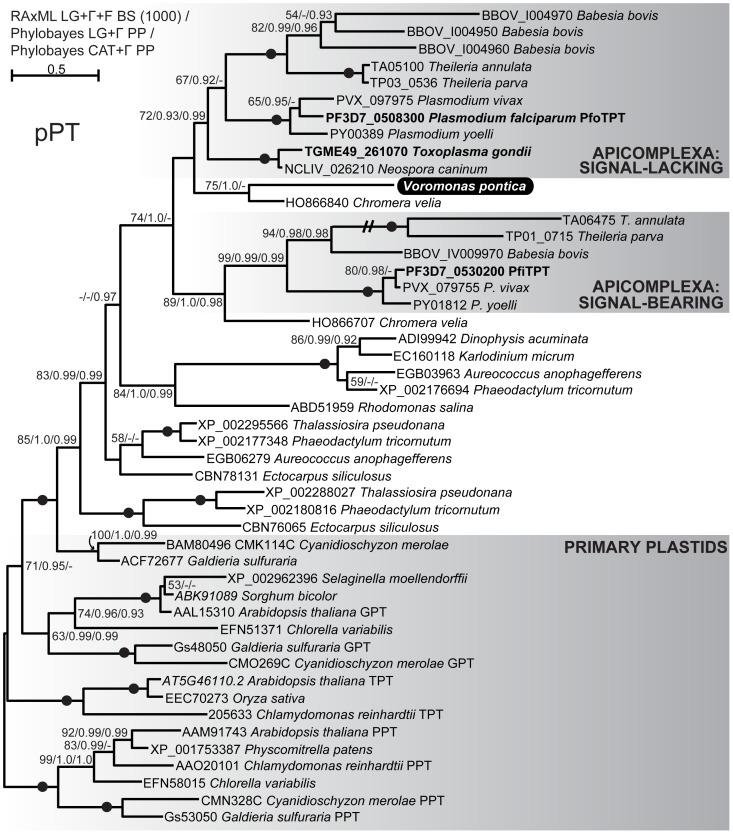
Maximum likelihood phylogeny of plastidic phosphate transporters. Support for nodes is indicated by % bootstrap support (out of 1000) in the ML analysis and by posterior probabilities from two Bayesian analyses, one employing the LG model of amino acids substitution, and the other using the CAT model (RAxML LG+Γ+F/Phylobayes LG+Γ/Phylobayes CAT+Γ), where greater than 50% bootstrap support or 0.9 posterior probability. Black dots on branches indicate full support from all three analyses for the adjacent node, i.e. 100/1.0/1.0. The subject of this study, *Voromonas pontica*, is indicated by white text on a black background. Experimentally apicoplast-localized proteins are indicated by bold text. Hatch marks indicate a branch whose length has been reduced by half. Aside from the primary plastid pPTs enclosed by the lower shaded box, all sequences belong to secondary, rhodophyte-derived plastids.

A homolog of secondary algal pPTs is present in our *V. pontica* transcriptomic data. The incomplete, 453 nt contig encodes 150 aa, approximately 45% of the mature protein, and is missing both the 5′ and 3′ ends. Although the N-terminus of the protein is not represented, we interpret the encoded pPT as a candidate plastid-targeted protein because it is clearly related to apicomplexan pPTs (Fig. 2), three of which have been experimentally localized, and because no pPTs are known from plastid-lacking organisms [47], [48].

### Phylogenetic analyses of putative plastid-associated proteins

**Figure 3:**
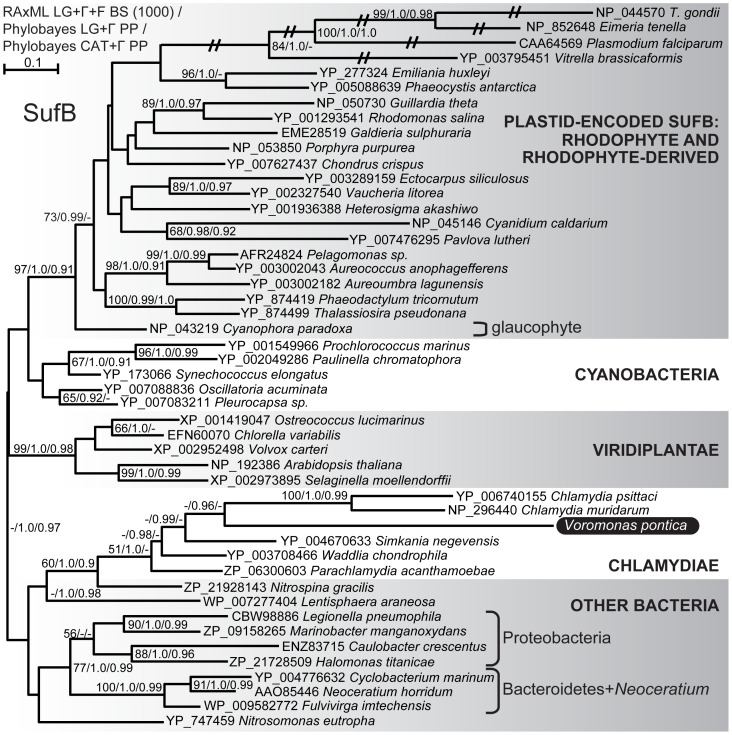
Maximum likelihood phylogeny of SufB amino acids sequences. Support for nodes is indicated by % bootstrap support (out of 1000) in the ML analysis and by posterior probabilities from two Bayesian analyses, one employing the LG model of amino acids substitution, and the other using the CAT model (RAxML LG+Γ+F/Phylobayes LG+Γ/Phylobayes CAT+Γ), where greater than 50% bootstrap support or 0.9 posterior probability. The subject of this study, *Voromonas pontica*, is indicated by white text on a black background. Hatch marks indicate branches whose lengths have been reduced by half.

We performed phylogenetic analyses of each of the plastid-associated genes found in the *V. pontica* partial transcriptome in order to a) confirm their BLAST-based identification, b) rule out potential contamination from *P. cosmopolitus* or bacteria present in the culture, and c) assess whether these proteins have undergone accelerated evolution relative to their homologs in other organisms. We used ML (RAxML) and Bayesian (Phylobayes) methods for all trees, and node support was assessed with bootstrap values and posterior probabilities, respectively. With the exception of SufB (Fig. 3, see discussion for details), all *V. pontica* sequences branch at least weakly (i.e. <50% bootstrap support, except higher for IspE and ALAS, <0.9 posterior probability except higher for IspE, ALAS, and ALAD) with apicomplexans and/or *P. marinus*, and therefore we expect they derive from *V. pontica* rather than contaminating bacteria or *P. cosmopolitus* (Fig. 2, Figs. S3, S4, S5, S6, S7, S8). Furthermore, we failed to see evidence for accelerated evolution in the *V. pontica* sequences as their branch lengths were always similar to or shorter than their functional alveolate homologs (Fig. 2, Figs. S3, S4, S5, S6, S7, S8).

### Targeting peptides in *V. pontica*

Four of the genes for putatively plastid-targeted proteins identified in this study are complete at the 5′ end and encode a predicted N-terminal signal peptide and putative transit peptide (TP) before the start of the conserved domain. Because TP cleavage sites are not well characterized, TP lengths were given as the number of amino acid residues between the signal peptide cleavage site and the start of the conserved domain alignments from NCBI's conserved domain database (Table 2, Fig. S2). This transit peptide length is likely to be an overestimate, in which case the computed amino acid frequencies would be affected by mature protein residues. As a comparison, therefore, we have measured the same characteristics for the first 20 residues of each transit peptide (Table 2). None of the four putative transit peptides in *V. pontica* bears a phenylalanine or tyrosine residue at the +1 position, in contrast to the transit peptides of other alveolates, particularly *C. velia* [44], [49].

## Discussion

### Phylogenetic position of *V. pontica*

Most previous phylogenetic analyses have placed colpodellids in a monophyletic group sister to apicomplexans, though only with moderate (i.e. 50–80% bootstrap) support [30], [33], [34]. However, in one poorly supported analysis, one colpodellid and a related environmental sequence instead branch sister to dinoflagellates while the remaining colpodellids form a grade at the base of the apicomplexans [30]. Later, Moore et al. [18] uncovered a sister relationship between the newly discovered photosynthetic *C. velia* and colpodellids; this clade then formed the sister lineage to apicomplexans, though neither of these relationships received strong support [18]. Two more recent studies found moderate support for a sister relationship between *C. velia* and apicomplexans, but no colpodellids were included [14], [50]. No single analysis has yet included sequences from colpodellids, *C. velia*, and the more recently discovered chromerid *V. brassicaformis*, and none has yet robustly resolved the relationships among colpodellids, chromerids, and apicomplexans. In order to address these uncertainties, we constructed a phylogeny of small subunit ribosomal RNA (SSU) sequences selected to cover the available phylogenetic diversity of apicomplexans, dinoflagellates, chromerids, and colpodellids. We chose to use SSU sequences because SSU is by far the most sampled gene for colpodellids, and we wished to place *V. pontica* in the most detailed, i.e. deeply sampled, evolutionary context possible. Increased taxon sampling can also improve phylogenetic resolution, especially in the absence of protein-coding sequence data [51], [52]. Protein sequences, by contrast, would be available for only *C. velia*, *V. brassicaformis*, and, from this study, *V. pontica*. We used Bayesian and maximum likelihood (ML) methods; the Bayesian topology with posterior probabilities and ML bootstrap values is shown in Fig. 1.

We can make only limited conclusions due to the lack of resolution in the currently available SSU data. We can, however, conclude that neither colpodellids nor chromerids are monophyletic, and we have moderate support for the hypothesis that *V. pontica* is more closely related to *C. velia* than to *V. brassicaformis*, apicomplexans, or dinoflagellates. Furthermore, the relationship of *C. velia* to the *Voromonas/Colpodella* clade and the separate branching of *V. brassicaformis* and the *Alphamonas* clade suggests that photosynthesis was lost at least twice in colpodellids. This point is in accordance with previous studies showing on one hand that *C. velia* and *V. brassicaformis* branch separately [14] and also that colpodellids and chromerids are related to the exclusion of apicomplexans [18], [30], [33], [34]. We were unable to determine whether chromerids and colpodellids together form a monophyletic group or whether one of the chromerid/colpodellid clades branches closer to the apicomplexans.

### Fe-S cluster biogenesis

Iron-sulfur (Fe-S) clusters are cofactors that help catalyze a variety of essential redox reactions in archaea, bacteria, and eukaryotes, including respiration, photosynthesis, and nitrogen fixation [53], [54]. In general, Fe-S clusters are assembled on a scaffold protein from sulfur cleaved from cysteine residues and iron donated by ferredoxin or another source before being transferred to the target apoprotein [55]. Four main Fe-S assembly systems have been characterized, each with its own cysteine desulfurase(s) and scaffold proteins. The ISC (iron sulfur cluster) system is the main system in bacteria and mitochondria, the SUF (sulfur mobilization) system is used by various bacteria under iron deficiency or oxidative stress conditions and is the main system in cyanobacteria and plastids, the NIF (nitrogen fixation) system is dedicated to providing Fe-S clusters to nitrogenase, and the CIA (cytosolic iron sulfur assembly) system is found in the cytoplasm of eukaryotes [54], [56]. Apicomplexans (not including gregarines or *Cryptosporidium*) are fairly typical of plastid-bearing eukaryotes in using the ISC, CIA, and SUF systems in their mitochondria, cytoplasm, and apicoplast, respectively [35]–[37], [57], [58]. The SUF system is critical for maintenance of the apicoplast [59].

The SUF system is thought to be ubiquitous in plastids (except perhaps in dinoflagellates ([60], though see also [61])), where it is required for assembling and depositing Fe-S clusters on essential plastid Fe-S proteins such as ferredoxin and the Rieske protein of cytochrome b6f [62]. Green algae and land plants encode plastid-targeted SUF proteins in their nuclei, while the majority of red algal and red algal-derived plastid genomes encode SufB and SufC in tandem [14], [54], [63]–[66]. Certain red-derived plastids no longer encode SufC, including apicomplexans, the haptophyte *Emiliania huxleyi* and pelagophytes *Aureococcus anophagefferens* and *Aureoumbra lagunensis* [14], [63], [67]–[73]. Currently, the only red-derived plastid genome shown to lack SufB is that of *C. velia* [14].

Using predicted apicoplast Fe-S cluster biogenesis proteins from *P. falciparum* and *Toxoplasma gondii* as queries, we searched for homologs in the *V. pontica* transcriptomic data (Table 1). The scaffold protein SufB is encoded in the apicoplast genome [63], [67], [68], [70], homologs of SufA, SufC, SufD, SufE, SufS, Nfu1, Fd, and FNR have been identified encoded in the nuclear genomes with bipartite targeting sequences to direct them to the apicoplast [35], [37], and ferredoxin, FNR, NFU, and SufC, SufE, and SufS have been experimentally localized [72]–[75]. Although plastid ferredoxin and its reductase FNR are better known for their roles in photosynthetic electron transport, they are the only known redox system in the apicoplast and are therefore thought to be indispensable to Fe-S biosynthesis [37]. We found clear *V. pontica* orthologs of SufB and SufS, but were unable to find orthologs of SufA, SufC, SufD, SufE, ferredoxin, or FNR. Due to the expected incompleteness of our transcriptome, it is unclear whether these genes are missing from *V. pontica* or simply missing from our data, but even among apicomplexans the complement of SUF components differs: SufA, SufB, SufC, SufD, and SufE are missing from the genomes of *Theileria parva* and *Babesia bovis*, despite the presence of SufS, Nfu1, Fd, and FNR [37].

In our phylogenetic analysis, the *V. pontica* SufB does not branch with the plastid-encoded SufB of apicomplexans and *V. brassicaformis*, rather, its closest relatives are chlamydiales bacteria (Fig. 3). The specific placement of *V. pontica* SufB is not supported, but other supported nodes separate it from the apicomplexans, strongly suggesting that the putatively plastid-targeted SufB in *V. pontica* was not acquired by endosymbiotic gene transfer, but rather by horizontal transfer, perhaps from a parasitic or endosymbiotic chlamydiales bacterium. While it is possible that the *V. pontica* SufB is actually a contaminating bacterial sequence, the presence of a predicted signal and putative transit peptide (Fig. S2) argue against this interpretation. It should be noted that SufB is missing from the plastid genome of *C. velia*, which our SSU phylogeny (Fig. 1) suggests is a close relative of *V. pontica*. If *C. velia* were found to encode a plastid-targeted SufB in its nucleus, it would be very interesting to see whether it was acquired from the same bacterial source, which would support the inference that *C. velia* and *V. pontica* share a common ancestor to the exclusion of *V. brassicaformis* and the apicomplexans, or an alternate source, suggesting either a functional endosymbiotic gene transfer or an independent horizontal transfer of SufB in *C. velia*.

The other SUF component we identified among the *V. pontica* transcripts, SufS, is a cysteine desulfurase related to NifS and IscS [76]. The distribution of SufS is markedly different from that of SufB. While SufB is encoded in most red and red-derived plastids, SufS is uncommon among these algae, and is never plastid-encoded. There is no SufS homolog from *Chondrus crispus* in NCBI NR, though the related mitochondrial iscS is present. Likewise, only 4 red-derived secondary algae have SufS orthologs in NR (*A. anophagefferens*, *Ectocarpus siliculosis*, *Phaeodactylum tricornutum*, and *Guillardia theta*); these are all included in Fig. S3. Also of note, SufS is the only Suf gene present in *Babesia* and *Theileria* [37]. Perhaps the apicoplast SUF system has taken an alternate evolutionary trajectory in this lineage of apicomplexans [37]. The topology of SufS is mainly unresolved in our phylogenetic analysis (Fig. S3); only the four clades of bacteria, land plants, green algae, and cyanobacteria are supported. The *V. pontica* sequence is excluded from the clades of bacteria, cyanobacteria, green algae, and land plants and shows a weak affinity for SufS from *T. gondii* and *P. marinus*, but is best considered unplaced.

### MEP pathway for isoprenoid biosynthesis

Isoprenoids, also known as terpenoids, form a large and diverse group of organic chemicals including carotenoids and steroids [77]. Isoprenoids are assembled from two basic building blocks, isopentyl diphosphate (IPP) and its isomer dimethylallyl diphosphate (DMAPP), both of which are synthesized via one of two distinct pathways, the mevalonate (MVA) pathway, and the non-mevalonate or methylerythretol pathway (MEP) [78]. The distribution of these two pathways appears to be quite complex in bacteria, where one, both, or neither pathway may be present with little regard to phylogenetic affinity. It is thought that HGT has played a major role in shaping the current distribution and phylogenetic relationships of isoprenoid biosynthesis genes, particularly in bacteria [79]. Generally, however, the MEP pathway is known from plastids while archaea and eukaryotes use the MVA pathway [78], [80]. The apicoplast MEP pathway has received considerable attention as a possible drug target [81], and two of the enzymes, ispC (DXR) and ispE, have been experimentally localized to the apicoplast in *P. falciparum* [82], [83], and one, ispH, to the apicoplast of *T. gondii* [84]. This pathway appears to be critical for parasite survival, as fosmidomycin, which inhibits ispC, is fatal to *P. falciparum* [85] and to *T. gondii* when engineered to express the appropriate transporter for uptake [84]. Interestingly, fosmidomycin-treated *P. falciparum* cells can be rescued by the addition of IPP, as can parasite cells whose apicoplasts have been ablated by treatment with other antibiotics, suggesting that isoprenoid biosynthesis is the *raison d'être* for the apicoplast, at least in blood-stage *P. falciparum* [86].

The MEP pathway has also been useful for unveiling cryptic plastids because it is found in the plastids of all investigated plastid-bearing eukaryotes except *Euglena gracilis* [80], [87], including non-photosynthetic plastid-bearing eukaryotes such as the alveolates *P. marinus*, *O. marina*, and *Crypthecodinium cohnii*, and the green algae *Prototheca wickerhamii* and *Helicosporidium* [6], [20], [21], [88]-[90]. Furthermore, the MEP pathway has yet to be found in any non-plastid bearing eukaryote [21], [80]; this is true not only of ancestrally non-photosynthetic organisms such as animals and fungi, but also of the secondarily plastid-lacking apicomplexan *Cryptosporidium* [91]. In *P. marinus*, 6 of the 7 enzymes were found, and a localization of ispC (DXR) permitted visualization of its cryptic plastid [21], [89].

We found genes for three MEP pathway enzymes in *V. pontica*, namely DXS, IspE, and IspG. None of the phylogenies are well resolved overall, but in all cases there is at least a weak affinity between *V. pontica* and one or more apicomplexans (Figs. S4, S5, S6). Therefore we expect these genes derive from *V. pontica* and are not contaminants. The relationship we found between chlamydia and eukaryotes for IspE and IspG genes has been noted previously [80], and is weakly to moderately supported in our phylogenetic analyses (Figs. S5, S6).

### Tetrapyrrole biosynthesis

Heme, chlorophyll, and cytochromes are examples of tetrapyrroles that illustrate the ubiquity and importance of this group of organic compounds. They are synthesized from δ-aminolevulinic acid (ALA) by a conserved pathway in all domains of life [92]. Synthesis of ALA, however, can occur in one of two ways. Glycine and succinyl-CoA can be combined and converted to ALA by ALA synthase (known as the C4 pathway), or glutamate from glutamyl-tRNA^Glu^ can be converted to ALA via glutamyl-tRNA reductase and glutamate-1-semialdehyde reductase (C5 pathway). The C4 pathway is known only from α-proteobacteria and mitochondria, while the C5 pathway is found in all other investigated bacteria, eukaryotes, and plastids [92], [93].

The distribution and subcellular location of heme biosynthetic reactions in eukaryotes depends on their nutritional status, or to be more precise, the presence or absence of a plastid (for excellent schematic representations, see ([94] or [95])). Plastid-lacking heterotrophs use the C4 pathway to make ALA from glycine and succinyl-CoA in the mitochondrion. Five subsequent biosynthetic steps take place in the cytosol, and their end product, protoporphyrinogen IX, is transported into the mitochondrion for two final steps culminating in protoheme. In photoautotrophs, the entire process, beginning with the C5 pathway for ALA synthesis, takes place in the plastid [93].

There are some interesting exceptions to this general rule, however. The excavate alga *E. gracilis* maintains two complete tetrapyrrole biosynthetic pathways: one in the mitochondrion and cytosol and another in the plastid [96], [97]. This fact has been invoked to explain the ability of *E. gracilis* to survive without its plastid [98], [99]. Apicomplexans and *C. velia* use hybrid pathways involving both the mitochondrion and the plastid. In both cases ALA is synthesized in the mitochondrion and then imported into the plastid. In *C. velia* the remainder of the pathway takes place in the plastid [95], while in apicomplexans, only the subsequent four steps (three in *T. gondii*) take place in the plastid [100], [101]. The sixth step, conversion of coproporphyrinogen III to protoporphyrinogen IX by coproporphyrinogen oxidase (CPOX) takes place in the cytoplasm, and protoporphyrinogen IX is imported into the mitochondrion for the final two steps [93], [101]. This pathway has been less well characterized in the dinoflagellate lineage. Genes for mitochondrially-targeted ALA synthase (ALAS) proteins have been detected in *P. marinus* and *O. marina* [95] but not core dinoflagellates [60]. Porphobilinogen deaminase (PBGD) may be plastid-targeted in *O. marina*, while ALA dehydratase (ALAD, also known as porphobilinogen synthase) appears to be cytosolic in *P. marinus* [20], [102].

Contigs encoding ALAS and ALAD were present in the *V. pontica* transcriptomic data, but homologs of the remaining enzymes were missing. We recovered a complete N-terminus for ALAS, which we propose represents a mitochondrial transit peptide even though our bioinformatically-based predictions are conflicting. Three other lines of evidence suggest the mitochondrion is the most likely location for the *V. pontica* ALAS. First, no eukaryotic ALAS protein has yet to be localized elsewhere than the mitochondrion [93]. Second, *in silico* subcellular localization prediction is known to be more accurate for well-studied groups of organisms such as animals and plants, and less accurate for more distantly related eukaryotes [103], [104]. Finally, the *V. pontica* ALAS has top blast hits to α-proteobacteria and the predicted mitochondrially-targeted *C. velia* and *P. marinus*, and likewise branches with the predicted mitochondrially-targeted ALAS sequences of *C. velia*, *P. marinus*, and *V. brassicaformis*, and the experimentally mitochondrion localized ALAS of *T. gondii* [100] in our phylogenetic analysis (Fig. S7). While it is possible that *V. pontica* relocated its mitochondrial-type ALAS to the cytoplasm, we think it more likely that this is a mitochondrially-targeted protein.

ALAD is also complete at the 5′ end and encodes a clearly predicted signal peptide followed by a stretch of 62 aa before the start of the conserved domain, with the characteristics of a plastid transit peptide. Our ML topology for ALAD placed *V. pontica* with plastid-targeted homologs of *C. velia* and apicomplexans within a Myzozoan clade with moderate (Fig. S8). Together, these observations suggest a genuine, plastid-targeted ALAD. Our inference of a mitochondrially targeted ALAS and a plastid-targeted ALAD in *V. pontica* is the same as the “hybrid” pathway predicted for apicomplexans and *C. velia*.

### Plastidic phosphate transporter

Whether photosynthetic or not, plastids must exchange metabolites with their hosts. Photosynthetic plant plastids typically export triosephosphates and 3-phosphoglycerate while nonphotosynthetic plant plastids import hexose phosphates. Both categories of plastid typically import phosphoenolpyruvate. All of this traffic is mediated by phosphate translocators, which permit the exchange of phosphorylated sugars for inorganic phosphate across the plastid membranes [105]. Plastidic phosphate transporters (pPTs) are monophyletic and derive from host endomembrane nucleotide sugar transporters [106]–[108]. Within the pPT clade, pPTs of secondary plastids of red algal origin are monophyletic and branch sister to red algal triosephosphate/phosphate antiporters [106], [108], [109].

In apicomplexans, the non-photosynthetic apicoplast exchanges inorganic phosphate for phosphoenolpyruvate, phosphoglyceric acid, and dihydroxyacetonephosphate via pPTs [47]. In *Plasmodium* there are two pPTs per species, one with an N-terminal signal and transit peptide and one without; in *P. falciparum* the signal-bearing pPT (called PfiTPT) has been localized to the inner apicoplast membrane while the signal-lacking pPT (PfoTPT) localizes to the outermost apicoplast membrane [110], [48]. However, in *T. gondii* there is only one pPT (called TgAPT1), which lacks N-terminal targeting information and localizes to multiple membranes [48], [111]. The genome of *Babesia bovis* encodes four distinct pPTs [48], while *Cryptosporidium*, which lacks a plastid, does not encode pPTs [48].

The pPT homolog found in *V. pontica* is clearly related to apicomplexan pPTs (Fig. 2), three of which have been experimentally localized, and no pPTs are known from plastid-lacking organisms [47], [48]. Two interesting points arise from this observation. First, the parallel branching order of apicomplexan and *C. velia* pPT subtypes suggests that the previously observed ancient duplication of this gene [48] in fact occurred before the divergence of *C. velia* and apicomplexans. Accordingly, the two distinct *C. velia* pPTs follow the pattern of *Plasmodium*: one copy (HO866707) bears an N-terminal signal peptide and branches with the signal-bearing *Plasmodium* homologs, and the other lacks a signal (HO866840) and branches with the signal-lacking homologs. Secondly, the *V. pontica* sequence is specifically related to the *C. velia* signal-lacking homolog, suggesting that this might therefore be an outer membrane-targeted pPT. This second point must be taken with caution, however, because the Bayesian CAT analysis failed to recover the topology displayed in Fig. 2, which was found in both Bayesian and ML LG analyses. Instead, the signal-lacking *C. velia* sequence and the *V. pontica* sequence branch sequentially at the base of the clade that includes dinoflagellates and the cryptophyte *Rhodomonas salina* (not shown), with low posterior probability values (0.8 and 0.78).

### Concluding remarks

As a step toward determining whether colpodellids retain a non-photosynthetic plastid, we generated and searched transcriptomic data from *V. pontica* for homologs of genes for apicoplast-targeted proteins. Here we interpret the presence of genes encoding homologs of apicoplast proteins as evidence that a non-photosynthetic plastid may be retained in *V. pontica*. However, without an experimental localization of these proteins, we do not know whether this is true. Given that *V. pontica* shares a photosynthetic ancestor with apicomplexans and dinoflagellates [14], the alternative interpretation is that the ancestral plastid was lost completely in this lineage. Such a scenario would not be without precedent: all lines of evidence point to a complete loss of plastid in *Cryptosporidium* [24]–[26]. If the ancestral plastid has been lost completely in *V. pontica*, the genes we found must either be nonfunctional, or their protein products function somewhere other than a plastid. Neither of these possibilities can be ruled out at present. Furthermore, this scenario would help explain why most of the plastid genes we sought were missing from the dataset.

Nevertheless, we consider the retention of a non-photosynthetic plastid to be the most reasonable interpretation of the data, for the following reasons. First, all of the putatively plastid-targeted enzymes from *V. pontica* (except the putatively horizontally transferred SufB) reveal at least a weak phylogenetic affinity for apicomplexan and/or chromerid homologs (Fig. 2, Figs. S3, S4, S5, S6, S7, S8). This suggests not only that they truly belong to *V. pontica* and are not bacterial or other eukaryotic contamination, but that their evolutionary rates have not been radically different. In no case did we encounter frameshift or nonsense mutations. Thus the genes and their phylogenies do not show evidence for accelerated evolution or decay, which might be expected for nonfunctional genes. Next, when complete at the 5′ end, all of the putative *V. pontica* genes for plastid-targeted proteins encode predicted signal peptides followed by transit-peptide like regions (Fig. S2), as do their apicoplast-targeted homologs. Thus we failed to find evidence that these plastid-associated proteins function in a different subcellular location. Finally, the absence of most plastid proteins may simply be due to the incompleteness of the dataset. The *V. pontica* transcriptome presented here has short average contig lengths (roughly 500 bp), a small number of unique sequences (roughly 11,000), and is missing approximately 40% of the expected core eukaryotic genes from CEGMA. Therefore we would expect a good portion of the genes for putatively plastid-targeted proteins also to be missing. Taken together, these lines of evidence point to the retention of a non-photosynthetic plastid in *V. pontica* as the better interpretation for the presence of plastid-associated genes.

To place the possibility of a retained, non-photosynthetic plastid in an evolutionary perspective, we performed a taxon-rich phylogenetic analysis of SSU sequences. Although the phylogeny was not completely resolved, we were able to infer that neither colpodellids nor chromerids are monophyletic (though together they may be a clade) and that photosynthesis was likely lost more than once in colpodellids (Fig. 1). Furthermore, the losses of photosynthesis in colpodellids were likely independent of the loss in apicomplexan parasites. Transcriptomic data (and experimental localizations) from other colpodellid lineages are needed to determine whether non-photosynthetic plastids may be present in other colpodellids, and if so, to infer the metabolic functions of independently reduced plastids. Colpodellids therefore represent a valuable source of comparative information for understanding the process of plastid reduction across different lifestyles (i.e. free-living heterotrophic vs. parasitic) but with similar genetic backgrounds.

## Materials and Methods

### Culture conditions, RNA extraction, and sequencing

*Voromonas pontica* ATCC 50640 was maintained at room temperature in vent-cap polystyrene flasks along with its prey, *Percolomonas cosmopolitus*. Every 10–20 days, 0.25 mL of the bi-eukaryote culture was transferred into 10 mL of bacterized ATCC medium 1525. In order to help identify contaminating *P. cosmopolitus* transcripts among the *V. pontica* data, a *P. cosmopolitus* mono-eukaryote culture was established from the bi-eukaryote culture by serial dilution and maintained in bacterized artificial seawater.

For transcriptome sequencing, 200 mL of *V. pontica* culture and 1500 mL of *P. cosmopolitus* culture were harvested by centrifugation. Total RNA was extracted with Trizol reagent (Invitrogen, Carlsbad, USA) according to the manufacturer's directions and quantified with a Qubit 2.0 fluorometer (Invitrogen, Carlsbad, CA, USA), yielding approximately 7 µg RNA from the bi-eukaryote culture and 200 µg RNA from *P. cosmopolitus*. All of the *V. pontica* and 20 µg of the *P. cosmopolitus* RNA were sent to the McGill University and Genome Quebec Innovation Centre for 100 bp paired-end Illumina library preparation and multiplex sequencing on a single lane of an Illumina HiSeq (Illumina, San Diego, CA, USA). 156 million reads were produced for *P. cosmopolitus* and 81 million reads for *V. pontica*.

Reads from each sample were trimmed using Trimmomatic [112] and assembled into 24,806 contigs for *V. pontica* and 11,374 contigs for *P. cosmopolitus* using Trinity [39] with minimum contig length set to 200 bp. Contaminating *P. cosmopolitus* contigs from the *V. pontica* library were identified by BLASTn and removed if 90% identical for at least 90% of the contig length using a perl script developed by David Morais at the McGill University and Genome Quebec Innovation Centre, leaving 13,970 filtered *V. pontica* contigs, including isoforms of the same locus (11,049 unique loci). Completeness of the transcriptome data set was estimated to be 70% using CEGMA [40], [41] at the 50% coverage level with expect cutoff of e^−10^.

### Data mining and targeting peptide characterization

Query files were assembled from *P. falciparum* and *T. gondii* proteins involved in apicoplast biosynthetic pathways, and tBLASTn was used to search the *V. pontica* filtered contigs. If a hit was not returned for the *P. falciparum* and *T. gondii* sequences, orthologs from algae or cyanobacteria were also used as queries. Pathways searched included the apicoplast SUF system for iron-sulfur cluster biosynthesis, the methylerythritol pathway (MEP) for synthesis of isoprenoids, the type II fatty acids synthesis pathway (FASII), and the heme biosynthesis pathway (Table 1). Putative plastid-targeted genes were also searched using a query file of experimentally localized apicoplast proteins downloaded from ApiLoc v. 3 (http://apiloc.biochem.unimelb.edu.au/apiloc).

Contigs with complete 5′ ends or successfully finished by 5′ RACE (see below) were conceptually translated and analyzed for the presence of signal peptides by signalP 4.0 [113]. Plastid transit peptides were inferred to begin at the signal peptide cleavage site, as determined by signalP 4.0, and to end at the last residue before the start of the conserved domain as determined by BLASTp against the NCBI conserved domains database [114]. Amino acid frequencies were computed using DNA Strider 1.43 [115] and the absence of stop-transfer transmembrane regions was determined using TMHMM [116]. SignalP and TMHMM were accessed via webservers of the Center for Biological Sequence analysis at the Technical University of Denmark. The mitochondrial transit peptide of ALAS was predicted with TargetP 1.1 [117], Euk-mPloc 2.0 [118], [119], and WoLF PSORT [120].

### Rapid amplification of cDNA ends (RACE)

Of the 7 transcripts encoding putatively plastid-targeted proteins we identified by BLAST, 4 were truncated at the 5′ end, and all were incomplete to varying degrees at the 3′ end. In order to characterize the N-termini of the encoded proteins, we performed 5′ RACE using the FirstChoice RLM-RACE kit (Ambion, Austin, TX, USA) using total RNA isolated using Trizol reagent (Invitrogen, Carlsbad, USA). In order to provide more characters for phylogenetic analyses, we characterized the 3′ ends of the most severely truncated transcripts with the same kit as for 5′ RACE. RACE PCR products were purified from TAE-buffered agarose gels using the UltraClean15 PCR purification kit (MoBio, Carlsbad, CA, USA) and ligated into the pCR 2.1 vector in The Original TA cloning kit with TOP10 competent cells (Invitrogen, Carlsbad, CA, USA). Vectors with inserts were purified from overnight cultures using the QuickClean 5 M Miniprep kit (Genscript, Piscataway, NJ, USA) and sent to Eurofins MWG Operon (Huntsville AL, USA) for Sanger sequencing on both strands. New sequences determined in this study were deposited in GenBank under accessions KF696859-66.

### Phylogenetic analyses

Small subunit ribosomal RNA sequences representing the diversity of dinoflagellates, apicomplexans, chromerids, and colpodellids were identified by BLAST and keyword searches, downloaded from the NCBI non-redundant (nr) database, and aligned with MAFFT [121]. Highly variable and ambiguously aligned sites were removed by eye using MacClade 4.08 [122] for a final alignment of 56 taxa and 1419 sites. Phylogenetic trees were inferred using maximum likelihood (ML) and Bayesian methods in the programs RAxML 7.0.4 [123], under the GTR+Γ model with four discrete rate categories, and PhyloBayes 3.2 [124], under the GTR+CAT model with four discrete rate categories. Support for ML topologies was assessed from 1000 bootstrap replicates. Bayesian analyses included two independent runs of 200,000 generations each, with one tree saved every 10 cycles. The first 5000 saved trees from each run were discarded as burn-in, and consensus trees and posterior probabilities were computed from the 30,000 pooled final trees from both runs. The maximum discrepancy across bipartitions between the two runs (maxdiff value) was 0.01.

Protein phylogenetic analyses also used MAFFT for alignment, MacClade for site trimming by eye, and RAxML and PhyloBayes for ML and Bayesian phylogenetic inference. The best-fit amino acids substitution model for each alignment was determined using prottest 3.2 [125], which preferred the LG model [126] with gamma-approximated rates (LG+Γ) for all alignments except SufB and TPT, which were better modeled by the inclusion of empirical amino acid frequencies (LG+Γ+F). Bayesian analyses included two independent chains run with the CAT model [127] and two independent chains using the prottest-specified model. For both Bayesian analyses, each chain was run for 25,000 generations, the first 5000 trees of each chain were discarded as burn-in, and the consensus tree and chain convergence statistics were computed from the total remaining 40,000 trees from both runs. Initial/final alignment lengths and maxdiff values for the LG/CAT Bayesian analyses are as follows. SufB: 587/466, 0.32/0.02; SufS: 820/381, 0.13/0.05; DXS: 1893/592, 0.08/0.06; ispE: 1385/194, 0.03/0.05; ispG: 705/439, 0.01/0.04; ALAS: 885/392, 0.00/0.03; ALAD: 748/317, 0.02/0.03; TPT: 650/261, 0.01/0.00. Alignments are available on request.

## Supporting Information

Figure S1 Maximum likelihood phylogeny of small subunit rRNA sequences from alveolates representing the available diversity of colpodellids, chromerids, and related environmental sequences. Support for nodes is indicated by % bootstrap support (out of 1000) in the ML analysis (RAxML GTRΓ)/Bayesian posterior probability (Phylobayes GTRCAT) where greater than 55 or 0.9. The subject of this study, *Voromonas pontica*, is indicated by white text on a black background. The photosynthetic chromerids *Chromera velia* and *Vitrella brassicaformis* are indicated by bold text. A question mark after accession AF372772 indicates a possible misidentification or chimeric sequence; this study also sampled a lake.
(PDF)

Figure S2 Characteristics of putative *V. pontica* N-terminal targeting peptides. A. Signal peptides predicted by SignalP 4.1. B. Putative bipartite plastid targeting sequences aligned at the predicted signal cleavage site (arrowhead). Amino acids are colored according to hydrophobicity and charge: yellow indicates hydrophobic residues (A, F, G, I, L, M, P, V), red indicates acidic residues (D, E), blue indicates basic residues (H, K, R), green indicates polar uncharged residues (C, N, Q, W, Y) with the exception of the hydroxylated residues serine and threonine (S, T) which are shown in purple. Putative transit peptides are shown as if cleaved directly before the start of the mature protein's conserved domain.
(PDF)

Figure S3 Maximum likelihood phylogeny of SufS amino acids sequences. Support for nodes is indicated by % bootstrap support (out of 1000) in the ML analysis and by posterior probabilities from two Bayesian analyses, one employing the LG model of amino acids substitution, and the other using the CAT model (RAxML LG+Γ/Phylobayes LG+Γ/Phylobayes CAT+Γ), where greater than 50% bootstrap support or 0.9 posterior probability. The subject of this study, *Voromonas pontica*, is indicated by white text on a black background.
(PDF)

Figure S4 Maximum likelihood phylogeny of DXS amino acids sequences. Support for nodes is indicated by % bootstrap support (out of 1000) in the ML analysis and by posterior probabilities from two Bayesian analyses, one employing the LG model of amino acids substitution, and the other using the CAT model (RAxML LG+Γ/Phylobayes LG+Γ/Phylobayes CAT+Γ), where greater than 50% bootstrap support or 0.9 posterior probability. The subject of this study, *Voromonas pontica*, is indicated by white text on a black background.
(PDF)

Figure S5 Maximum likelihood phylogeny of IspE amino acids sequences. Support for nodes is indicated by % bootstrap support (out of 1000) in the ML analysis and by posterior probabilities from two Bayesian analyses, one employing the LG model of amino acids substitution, and the other using the CAT model (RAxML LG+Γ/Phylobayes LG+Γ/Phylobayes CAT+Γ), where greater than 50% bootstrap support or 0.9 posterior probability. The subject of this study, *Voromonas pontica*, is indicated by white text on a black background.
(PDF)

Figure S6 Maximum likelihood phylogeny of IspG amino acids sequences. Support for nodes is indicated by % bootstrap support (out of 1000) in the ML analysis and by posterior probabilities from two Bayesian analyses, one employing the LG model of amino acids substitution, and the other using the CAT model (RAxML LG+Γ/Phylobayes LG+Γ/Phylobayes CAT+Γ), where greater than 50% bootstrap support or 0.9 posterior probability. The subject of this study, *Voromonas pontica*, is indicated by white text on a black background.
(PDF)

Figure S7 Maximum likelihood phylogeny of ALAS amino acids sequences. Support for nodes is indicated by % bootstrap support (out of 1000) in the ML analysis and by posterior probabilities from two Bayesian analyses, one employing the LG model of amino acids substitution, and the other using the CAT model (RAxML LG+Γ/Phylobayes LG+Γ/Phylobayes CAT+Γ), where greater than 50% bootstrap support or 0.9 posterior probability. The subject of this study, *Voromonas pontica*, is indicated by white text on a black background.
(PDF)

Figure S8 Maximum likelihood phylogeny of ALAD amino acids sequences from photosynthetic organisms. Chlamydiae were included as the closest non-photosynthetic outgroup of the cyanobacterial and plastid clade; other bacteria, archaea, and the cytoplasmic proteins of eukaryotes branch separately [95]. Support for nodes is indicated by % bootstrap support (out of 1000) in the ML analysis and by posterior probabilities from two Bayesian analyses, one employing the LG model of amino acids substitution, and the other using the CAT model (RAxML LG+Γ/Phylobayes LG+Γ/Phylobayes CAT+Γ), where greater than 50% bootstrap support or 0.9 posterior probability. The subject of this study, *Voromonas pontica*, is indicated by white text on a black background.
(PDF)
