# Supplementary material for: Transcriptomic Analysis Reveals Evidence for a Cryptic Plastid in the Colpodellid Voromonas pontica, a Close Relative of Chromerids and Apicomplexan Parasites
Source: PLoS One. 2014 May 5;9(5):e96258. doi: 10.1371/journal.pone.0096258 (PMC4010437; doi:10.1371/journal.pone.0096258)
Supplement: Figure S1 — Maximum likelihood phylogeny of small subunit rRNA sequences from alveolates representing the available diversity of colpodellids, chromerids, and related environmental sequences. Support for nodes is indicated by % bootstrap support (out of 1000) in the ML analysis (RAxML GTRΓ)/Bayesian posterior probability (Phylobayes GTRCAT) where greater than 55 or 0.9. The subject of this study, Voromonas pontica, is indicated by white text on a black background. The photosynthetic chromerids Chromera velia and Vitrella brassicaformis are indicated by bold text. A question mark after accession AF372772 indicates a possible misidentification or chimeric sequence; this study also sampled a lake. (PDF) [file pone.0096258.s001.pdf]

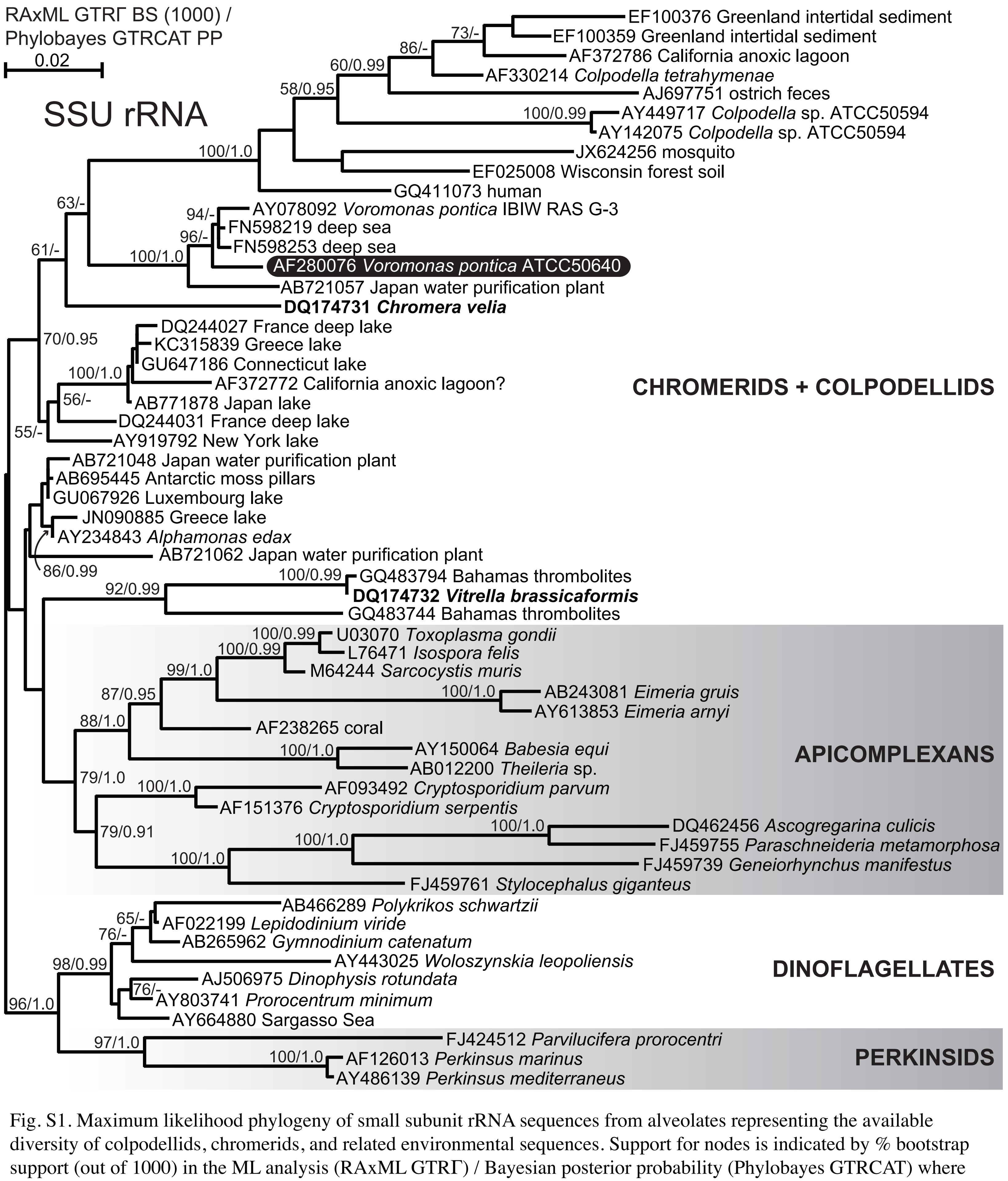

Fig. S1. Maximum likelihood phylogeny of small subunit rRNA sequences from alveolates representing the available diversity of colpodellids, chromerids, and related environmental sequences. Support for nodes is indicated by % bootstrap support (out of 1000) in the ML analysis (RAxML GTRΓ) / Bayesian posterior probability (Phylobayes GTRCAT) where greater than 55 or 0.9. The subject of this study, *Voromonas pontica*, is indicated by white text on a black background. The photosynthetic chromerids *Chromera velia* and *Vitrella brassicaformis* are indicated by bold text. A question mark after accession AF372772 indicates a possible misidentification or chimeric sequence; this study also sampled a lake.
