# Supplementary material for: Transcriptomic Analysis Reveals Evidence for a Cryptic Plastid in the Colpodellid Voromonas pontica, a Close Relative of Chromerids and Apicomplexan Parasites
Source: PLoS One. 2014 May 5;9(5):e96258. doi: 10.1371/journal.pone.0096258 (PMC4010437; doi:10.1371/journal.pone.0096258)
Supplement: Figure S2 — Characteristics of putative V. pontica N-terminal targeting peptides. A. Signal peptides predicted by SignalP 4.1. B. Putative bipartite plastid targeting sequences aligned at the predicted signal cleavage site (arrowhead). Amino acids are colored according to hydrophobicity and charge: yellow indicates hydrophobic residues (A, F, G, I, L, M, P, V), red indicates acidic residues (D, E), blue indicates basic residues (H, K, R), green indicates polar uncharged residues (C, N, Q, W, Y) with the exception of the hydroxylated residues serine and threonine (S, T) which are shown in purple. Putative transit peptides are shown as if cleaved directly before the start of the mature protein's conserved domain. (PDF) [file pone.0096258.s002.pdf]

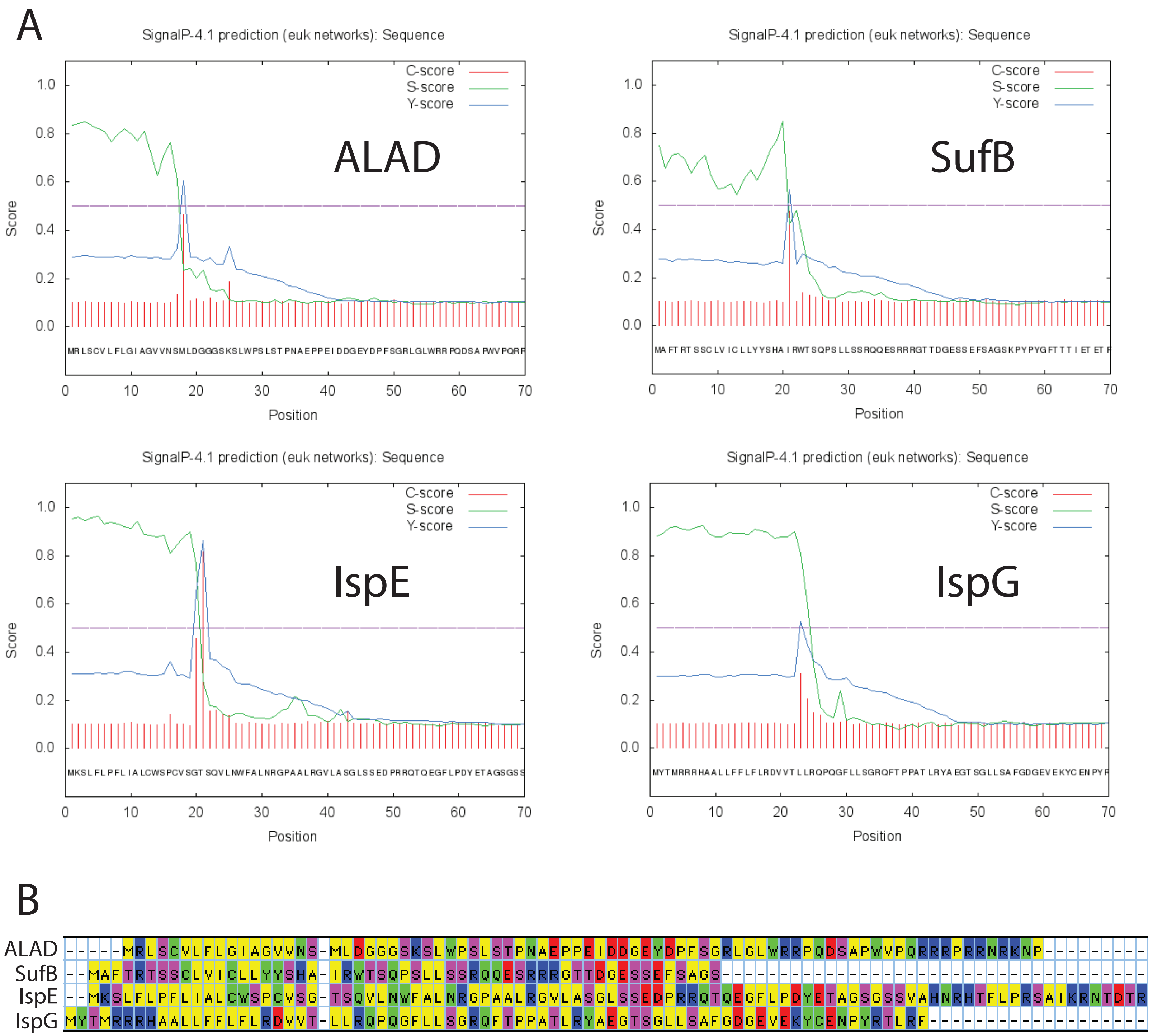

Fig. S2. Characteristics of putative *V. pontica* N-terminal targeting peptides. A. Signal peptides predicted by SignalP 4.1. B. Putative bipartite plastid targeting sequences aligned at the predicted signal cleavage site (arrowhead). Amino acids are colored according to hydrophobicity and charge: yellow indicates hydrophobic residues (A, F, G, I, L, M, P, V), red indicates acidic residues (D, E), blue indicates basic residues (H, K, R), green indicates polar uncharged residues (C, N, Q, W, Y) with the exception of the hydroxylated residues serine and threonine (S, T) which are shown in purple. Putative transit peptides are shown as if cleaved directly before the start of the mature protein's conserved domain.
