# Supplementary figures and images for: Transcriptomic Analysis Reveals Evidence for a Cryptic Plastid in the Colpodellid Voromonas pontica, a Close Relative of Chromerids and Apicomplexan Parasites
Source: PLoS One. 2014 May 5;9(5):e96258. doi: 10.1371/journal.pone.0096258 (PMC4010437; doi:10.1371/journal.pone.0096258)

# ALAS

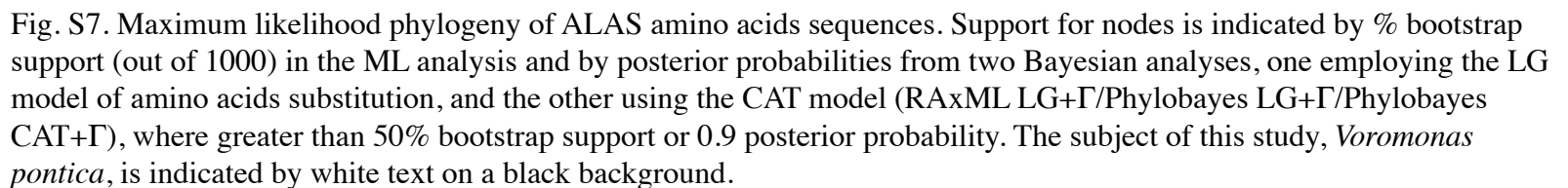

Supplement: Figure S7 — Maximum likelihood phylogeny of ALAS amino acids sequences. Support for nodes is indicated by % bootstrap support (out of 1000) in the ML analysis and by posterior probabilities from two Bayesian analyses, one employing the LG model of amino acids substitution, and the other using the CAT model (RAxML LG+Γ/Phylobayes LG+Γ/Phylobayes CAT+Γ), where greater than 50% bootstrap support or 0.9 posterior probability. The subject of this study, Voromonas pontica, is indicated by white text on a black background. (PDF) [file pone.0096258.s007.pdf]
